# Crème de la Créature: Dietary Influences on Behavior in Animal Models

**DOI:** 10.3389/fnbeh.2021.746299

**Published:** 2021-09-29

**Authors:** Manaswini Sarangi, Monica Dus

**Affiliations:** Department of Molecular, Cellular, and Developmental Biology, The University of Michigan, Ann Arbor, MI, United States

**Keywords:** diet-induced obesity, animal models, high fat and high sugar diet, nutrition, behavior

## Abstract

In humans, alterations in cognitive, motivated, and affective behaviors have been described with consumption of processed diets high in refined sugars and saturated fats and with high body mass index, but the causes, mechanisms, and consequences of these changes remain poorly understood. Animal models have provided an opportunity to answer these questions and illuminate the ways in which diet composition, especially high-levels of added sugar and saturated fats, contribute to brain physiology, plasticity, and behavior. Here we review findings from invertebrate (flies) and vertebrate models (rodents, zebrafish) that implicate these diets with changes in multiple behaviors, including eating, learning and memory, and motivation, and discuss limitations, open questions, and future opportunities.

## Introduction

In the span of a few generations, shifts in economic, manufacturing, trading, and urban policies have profoundly reshaped the social and physical spaces where we eat, as well as the food choices available to us. Foodstuff today is generally “more”: more affordable, more accessible, and more safe compared to what our grandparents ate; it is also calorically-dense, larger in portion size, and with higher levels of salt, refined sugar, and saturated fat. These changes in diet composition have contributed to a higher incidence of noncommunicable diseases and a lowered life expectancy worldwide; they have also influenced brain physiology and behavior. In humans, consumption of diets with high levels of saturated fat and refined sugars is associated with impairments in memory performance, mood regulation, sensations, and motivated behaviors, and linked to an increased risk of neurological disorders such as Alzheimer’s, dementia, Parkinson’s, depression, and anxiety. Although studies have reported broad differences in inflammation, oxidative stress, neurochemicals, and neurogenesis with energy-dense diets and high body mass index (BMI; Luchsinger et al., 2002; López-Taboada et al., 2020), the specific molecular, cellular, and neural mechanisms remain to be discovered, and with them interventions that prevent or treat these diseases. To find answers researchers have turned to animal models. There are clearly aspects of neural function and behavior that can only be studied when we, humans, are the model. However, research on vertebrate and invertebrate model organisms has uncovered neural and molecular mechanisms that implicate diet composition—specifically high fat and sugar—with behavioral and neurophysiological dysfunctions. In this review, we highlight their findings, discuss lessons learned, and identify questions and opportunities that remain open.

## Modeling Dietary Influences on Behavior in Animals

*“[Model organisms] are Nature’s gift to Science” Sidney Brenner, Nobel Lecture*

**Figure 1:**
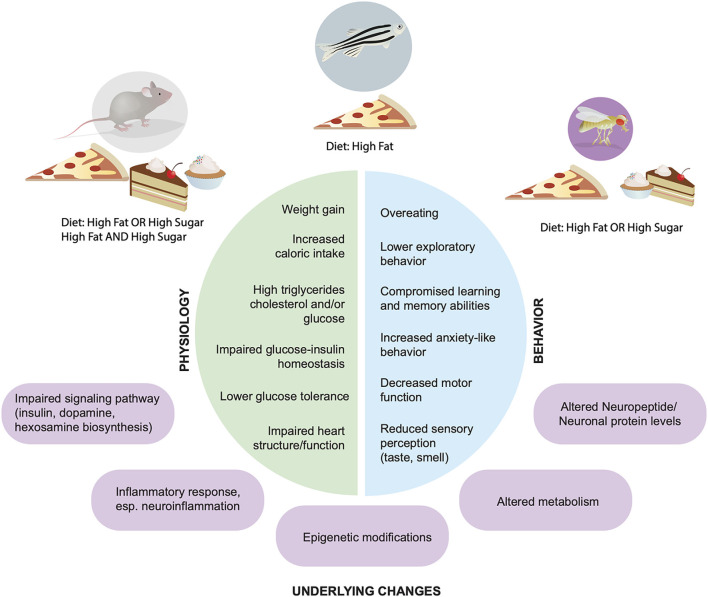
Physiological and behavioral effects of high fat and sugar diets. Across animal models *Rattus norvegicus*, *Mus musculus*, *Danio rerio*, *Drosophila melanogaster* (top) dietary manipulations lead to alterations in physiology (left, green) and behaviors (blue, right); underlying molecular and cellular mechanisms are shown in purple.

**Figure 2:**
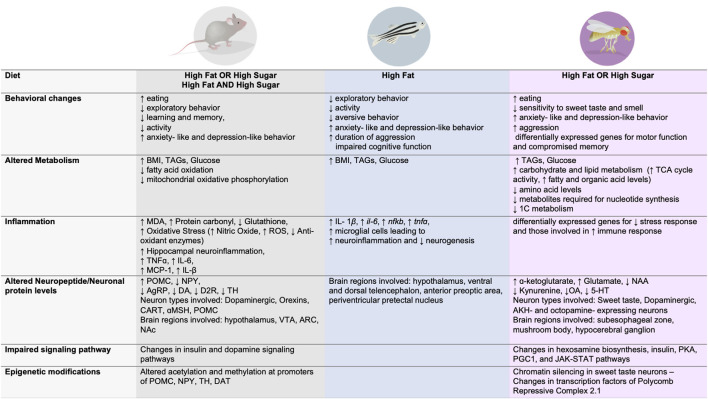
Dietary effects on behavior, metabolism, immune function, signaling, neuronal changes, and epigenetic modifications across three animal model systems.

In 1929 Nobel winner and physiologist August Krogh famously wrote that “For a large number of problems there will be some animal of choice, or a few such animals, on which it can be most conveniently studied.” Krogh’s principle has held true for almost a century: the use of model organisms has stimulated the development of tools, surmounted limitations, and driven innovation and discoveries that have led to treatments in all fields of biology, from genetics and molecular biology, to physiology, immunology, and neuroscience. A variety of model organisms have also been employed to study nutrition and the effect of diet composition on metabolic and brain health. Large mammalian models, like cats, dogs, and swine, as well as non-human primates, such as rhesus macaques, have been used in dietary studies, especially those related to diet-induced obesity and type-2 diabetes, thanks to their similar anatomy, physiology, and metabolism; the effects of seasonal dietary variation in natural habitats have also been investigated in large mammals such as seals (Chatzigeorgiou et al., 2009; Suleiman et al., 2020). However, besides the ethical considerations, the large size of these models, their cost, and the lack of genetic tools has prompted the adoption of animal models that are smaller, cost-effective, and tractable for mechanistic studies. Among these, laboratory mice and rats have been the most used, but more recently the effects of high fat and high sugar diets have also been modeled and examined in zebrafish and flies. This review will focus on evidence that has emerged from studies in these four laboratory animal models (Figures 1, 2).

In similar ways as with humans, high fat and/or high sugar diets promote overconsumption, an increase in fat mass and body weight, as well as alterations in metabolism and energy homeostasis in rodents, fish, and flies (Figures 1, 2). Importantly, all of these experimental animals show an increase in food intake and often larger, longer, or more frequent meals in response to energy-dense diets, although across animals there is an urgent need to standardize diet composition, exposure, and environmental influences (i.e., temperature, cage location, etc). However, each model has unique advantages and disadvantages when it comes to studying the impact of these diets on the brain and behavior. Mice and rats, the animals that have been most widely studied in this context, are considered the gold standard in preclinical research today because they are omnivorous mammals with similar physiology, neuroendocrinology, and overall nervous system and behavioral complexity to humans, which are ideal features for identifying new therapeutics. Tools that label, record, visualize, trace, and manipulate neural activity are also well established in mice and emerging in rats, which provide a way to link diet, behavior, and neural function. However, rodents are expensive to maintain and exposures to diet are lengthy (many months), which limits the sample size and different dietary conditions that can be tested. Further, manipulating the levels of genes to test necessity and sufficiency is also hard to do systematically in mice and rats, hindering mechanistic studies and the identification of disease-risk genes. Finally, uncoupling dietary exposure from its physiological effects has been challenging, as most diets promote overt obesity and metabolic syndrome, leaving questions about the etiology of neural and behavioral changes largely unanswered.

In contrast, mechanistic and genetic studies are easily accessible in zebrafish and flies, thanks to their fast generation time, high fecundity, transgenic gene expression tools, small size, low-rearing cost, and genetic similarity to humans (84% and 77% of diseases genes are conserved, respectively, compared to 90% in mice). In addition to being exquisite genetic models, flies and zebrafish have a rich repertoire of both simple and complex behaviors that can be measured and characterized in response to diet and genetic manipulations, as well as tools to visualize, manipulate, and label neural circuits (especially in developing animals), and perform genetic rescues. Because zebrafish are vertebrates, they show high anatomical conservation with the brain and organs of mammals, especially in terms of neuroendocrine regulation; this has made them exceptional experimental models for neurodevelopment and for drug screens. In contrast, some of the brain regions and organs of flies only show functional, not anatomical conservation, although they do share common genetic specification and identity programs that determine their properties, such as connectivity, organization, or gene expression; importantly, some neuroendocrine pathways critical for energy homeostasis regulation are absent in flies, like the melanocortin or the agouti-related peptide pathways. On the other hand, the simple brain of flies where a connectome for several brain regions is published or in progress, together with the constellation of publicly available and cost-effective transgenic resources, make this model organism ideal to dissect and uncouple the molecular and circuit mechanisms through which diet composition influences brain and behavior differently from body/fat mass and/or metabolic syndrome. Thus, answering questions in the neuroscience of nutrition field will benefit from applying Krogh’s principle: exploiting the tools and advantages of each experimental organism and integrating knowledge across them to understand common principles as well as differences. In the sections below we review studies in each model, consider what these findings have taught us, and discuss how this will shape future research on the role of nutrition in directing neurophysiology and behavior.

## The Effects of High Fat And/Or High Sugar Diets on Rodent Behaviors

*“If you are what you eat, then I only want to eat the good stuff.”*

*– Little Chef Remy from Ratatouille©*

Several types of feeding regimens are used to induce diet-induced obesity and mimic the composition of modern human diets in rodents: the most common is to provide animals with chow that contains higher levels of fat and/or sugar, or to supplement a diet of standard chow with fats and sugar in the form of lard, sucrose, or fructose, or human food such as biscuits, cereal, and chocolate chip cookies (the latter are often called “junk-food” or “cafeteria” diets in the literature; Hurley and Moran, 2020). These dietary manipulations are thought to recapitulate the high levels of saturated fats and refined sugars found in processed food common to industrial countries (and thus originally labeled “Western style”), although consumption of these foods is now common worldwide. Despite the ability to control the type and amount of food consumed, this diversity in dietary regimens, as well as the differences in duration of dietary exposure, genetic background, and rearing conditions, can result in substantial variation in phenotypes and outcomes, as reviewed in (Ellacott et al., 2010; Seeley and MacDougald, 2021). Because of this, it is important to carefully consider these factors when comparing and extrapolating across different studies even in the same model. *R. norvegicus* and *M. musculus* have been the most widely studied species, but dietary manipulations have also been performed in other rodent models, such as the Israeli sand rat, spiny mice, tuco tuco rats, and hamsters (Suleiman et al., 2020), although these will not be covered in this review. Of note, rats and mice that are obesity-prone or obesity-resistant (Madsen et al., 2010; Ferrario, 2020), as well as genetically-obese animals (Kleinert et al., 2018), have also been used to study behavioral susceptibilities to energy-dense dietary environments.

In rats and mice, the concentration of fat and the length of time to which animals are exposed to the diet vary greatly, but typically these are in the range of 40–60% fat and last a few weeks to several months (1–52 weeks, with 2–4 weeks and 3 or 6 months being more common). Longer exposures (<3–6 months) lead to higher calorie intake and body weight, and are associated with a large number of physiological alterations, including hyperglycemia, hyperinsulinemia, and dyslipidemia (triglycerides and cholesterol), heart and endothelial dysfunctions, liver and kidney fibrosis, and dysregulated immune responses, most of which are hallmarks of metabolic syndrome in humans (Figures 1, 2). A diet high in fat or high in both fat and simple carbohydrates is also linked to higher oxidative stress biomarkers, such as malondialdehyde, reactive oxygen species, and protein carbonyls in the liver, heart, and kidneys, in addition to lower levels of glutathione, antioxidant enzymes, and the mitochondrial quinone pool (Noeman et al., 2011; Panchal et al., 2011; Vial et al., 2011; Lozano et al., 2016). Supplementation of the standard chow diet with sugars like sucrose or fructose in the drinking water (ranging from 10 to 55%) has similar effects to the high fat diet over longer exposures, including higher calorie intake, fasting blood glucose, insulin, and leptin levels, and fat stores, but no changes in high-density cholesterol levels (Jurdak et al., 2008; Beilharz et al., 2014; la Fleur et al., 2014; Kothari et al., 2017). Many different behaviors have been phenotyped in response to dietary manipulations in rodents, including feeding and reward, cognition, chemosensation and sleep; below we focus primarily on the first two, as the relationship between diet and chemosensory plasticity (Peng et al., 2019; May et al., 2020; Reed et al., 2020) and diet and sleep (Frank et al., 2017) have been recently reviewed.

### Ingestive Behavior

Early studies in Sprague Dawley rats discovered that giving animals human food that was rich in sugar and fat resulted in higher food intake –quantified through the duration and number of meals– lower acceptance of lower calorie diets, and decreased wheel-running activity (Levitsky and Collier, 1968; Sclafani and Springer, 1976; Chandler et al., 2005); interestingly, high fat diets were less satiating and produced higher calorie intake than high carbohydrate diets in rats (Warwick and Weingarten, 1995). To better model human exposure to fatty and sugary food, one study provided male Wistar rats with high fat and high sugar foods in addition to chow, rather than only feeding them with chow that was high in fat and sugar. These rats showed persistent hyperphagia with larger but fewer meals, as well as higher motivated behaviors compared to animals that were not given a choice; interestingly, supplying both of these groups with high concentrations of sucrose in their drinking water increased meal number (la Fleur et al., 2014). Chronic high fat feeding for 12 weeks also hindered the responses of lateral hypothalamic glutamate-expressing neurons to sucrose; activity of this circuit is thought to inhibit feeding after meals, and indeed, the high fat diet fed rats ate more and accumulated fat (Rossi et al., 2019). These changes in meal size and number suggest that the processes of satiation and satiety may be altered (Chambers et al., 2013), something that has been measured in people given ultra processed food diets (Hall et al., 2019).

Satiation involves the association between sensory cues and the properties of food, and indeed rodents fed high fat diets show impairments in food reward learning (Davidson et al., 2014). Changes in sensory perception, primarily taste and smell have been observed with high BMI and with diet composition in humans and animals (Peng et al., 2019; May et al., 2020). In mice and rats, consumption of high fat, high sucrose, or a mixture of the two, dulled the responses of the taste-receptor expressing cells, the sensory nerves, and the Nucleus of the Solitary Tract to sweetness, with correspondingly alterations in taste receptor and signal transduction effectors (Maliphol et al., 2013; Weiss et al., 2019; Ahart et al., 2020; McCluskey et al., 2020); other groups have also observed lower renewal of taste cells with obesity (Kaufman et al., 2020). In mice, a high fat diet also blunted olfactory responses (Takase et al., 2016), olfactory discrimination, and promoted the loss of olfactory neurons and their axons (Thiebaud et al., 2014). These alterations in chemosensation could impair reward learning, a crucial component of satiation, and interestingly, defects in cue-reward learning have been observed in rodents fed high fat or human processed food diets (Swithers and Davidson, 2008; Davidson et al., 2014; Thiebaud et al., 2014; Lietzau et al., 2020).

The processing of the nutrient and hormonal signals that modulate satiety is also influenced by diet, and numerous studies found that rodents fed high fat diets were less sensitive to these signals. Specifically, rats and mice fed a high fat diet had lower responses to intraperitoneal Cholecystokinin (CCK) injection—a satiety peptide released in response to food influx into the duodenum—(Covasa and Ritter, 1998; Savastano and Covasa, 2005; Nefti et al., 2009); this decrease correlated with the amount of food ingested (Savastano and Covasa, 2005) and with the levels of CCK-receptor in the vagal afferents (Nefti et al., 2009). Responses to other satiety signals, such as the peptide bombesin and the satiety-inducing nutrient oleoylethanolamine, were also desensitized in rats and mice on a in high fat diet (Covasa and Ritter, 1998, 2000; Tellez et al., 2013). High fat feeding was also shown to elevate the anorexigenic leptin and POMC peptides/hormones, and to lower the orexigenic hypothalamic NPY and Agouti-related peptide (AgRP) peptides (Ziotopoulou et al., 2000); further, it changed the levels of DNA methylation and histone modifications at these gene loci (Marco et al., 2013, 2016; Cifani et al., 2015; Lazzarino et al., 2017). The responses of the AgRP neurons to food were also blunted by consumption of a high fat diet, and these phenotypes persisted even after animals were switched to a diet of normal chow and lost the excess weight (Beutler et al., 2020); of note, a high fat diet also altered the extrinsic and intrinsic excitability of these neurons (Vernia et al., 2016; Paeger et al., 2017; Korgan et al., 2021). Further, the number of cells expressing the anorexigenic cocaine and amphetamine-regulated transcripts (CART) and the mRNA levels of the α-melanocyte-stimulating hormone (α-MSH) in the hypothalamus were also decreased (Huang et al., 2003; Tian et al., 2004); neuroepigenetics changes in response to calorie-dense food environments were recently reviewed in Vaziri and Dus (2021).

More recently, studies have begun to investigate the effects of palatable, high-energy diets on the reward system, drawing parallels between natural rewards and drugs of abuse. Circuitry in the mammalian mesolimbic area plays a critical role in the processing of natural reinforcers and in assigning values to the sensory cues that become associated with them during learning. Exposure to energy-dense diets leads to adaptations in this neurocircuitry that are thought to reshape both the saliency, desire, and pleasure derived from food, as well as the responses to food cues (DiFeliceantonio and Small, 2018; Janssen et al., 2019); together these factors could contribute to higher intake and lower satiation and satiety. Indeed, in rats consumption of food high in both fat and sugar led to compulsive responses to food and higher thresholds for reward receipt (Johnson and Kenny, 2010) exposure to a high fat diet in mice devalued standard chow and its ability to alleviate hunger even after the animals were deprived from the high fat diet for 2 weeks (Mazzone et al., 2020). Further, animals given human foods high in fat and sugar showed impaired sensory cue-induced satiety (Reichelt et al., 2014) and reinforcement learning in both pavlovian and operant conditioning paradigms (Kendig et al., 2013; Davidson et al., 2014). A key neurotransmitter that modulates these behaviors is dopamine, and interestingly, high fat and sugar, in combination or alone, decreased the mRNA and protein levels of striatal Dopamine 2 Receptor (D2R) in both outbred and obesity-prone and -resistant rats and mice (Huang et al., 2006; Johnson and Kenny, 2010; Robinson et al., 2015; Jones et al., 2017; Barry et al., 2018; Rospond et al., 2019). Blunted dopamine transmission in response to intra-gastric infusion of lipids was also observed in mice fed a high fat diet for 4 months (Tellez et al., 2013). Of note, changes in Dopamine Receptor 2 binding, and dopamine signaling more broadly, have also been described in people with obesity (Janssen et al., 2019), as well as changes in reward learning and cue reactivity (Stice et al., 2011; Kroemer and Small, 2016). However, whether these differences are a cause or consequence of body weight in rodents or humans is still unclear, as some changes were observed with diet composition alone (Janssen et al., 2019). Adaptations in the rodent dopamine system with diet-induced obesity are not limited to dopamine receptors, and many studies have also reported changes in dopamine synthesis, reuptake, and release (Rada et al., 2010; Kessler et al., 2014; Jones et al., 2017; Patel et al., 2019). In particular, lower mRNA expression and higher DNA methylation of the tyrosine hydroxylase (TH) gene—the rate limiting enzyme in the synthesis of dopamine—was measured in the brains of mice and rats exposed to high fat and high sugar diets (Li et al., 2009; Lee et al., 2010; Vucetic et al., 2012; Robinson et al., 2015), and lower dopamine reuptake and evoked release were observed in rats fed a high fat diet for 6 weeks in the absence of obesity (Cone et al., 2013).

### Learning and Memory

Besides effects on ingestive and motivated behaviors, consumption of calorie-dense diets have also been linked to cognitive dysfunctions in humans (Yeomans, 2017). Indeed, high intake of saturated fat is associated with memory decline and a lower speed and flexibility in carrying out tasks (Ortega et al., 1997; Kalmijn et al., 2004; Morris et al., 2004; Okereke et al., 2012), a higher risk of dementia in individuals older than 55 years (Kalmijn et al., 1997; Morris et al., 2003), and an increase in attention deficits (Edwards et al., 2011; Holloway et al., 2011). To understand the role diet composition plays in these conditions and uncover the underlying neural and molecular mechanisms, researchers have tested the effects of dietary manipulations on learning and memory using several assays in mice and rats (Kanoski and Davidson, 2011; Cordner and Tamashiro, 2015).

Young, male Long Evans rats fed a diet supplemented with lower levels of polyunsaturated or saturated fats (20% lard or soybean oil or lard) showed impairments in spatial and temporal memory characterized by little to no improvement in learning over time; these deficits were mild in the soybean oil (polyunsaturated fats) but evident in the lard-fed rats compared to controls (Greenwood and Winocur, 1990, 1996). Interestingly, providing rats on these high fat diets with a socially enriched environment almost entirely rescued the learning and memory deficits (Winocur and Greenwood, 1999). Deficits in place learning as measured via the Morris Water Maze have also been uncovered with exposure to diets composed of different levels of fat and sugar, suggesting that the hippocampal and cortical circuitry are affected (Molteni et al., 2002; Wu et al., 2003; Molteni et al., 2004; Goldbart et al., 2006; Farr et al., 2008; Jurdak et al., 2008; Stranahan et al., 2008; Yu et al., 2010). The idea that the high fat and high sugar alter the working of the hippocampus and the cortex is also supported by studies that used other assays to show impairments in working and spatial memory in rats on diets high in fat or both fat and sugar (Greenwood and Winocur, 1990; Murray et al., 2009; Kanoski and Davidson, 2010; Boitard et al., 2014), rats on just a high sucrose diet (Jurdak et al., 2008) or a fed human food high in fat and sugar (Beilharz et al., 2014), and mice on a high fat diet (Valladolid-Acebes et al., 2011; Arnold et al., 2014). However, deficits in non-spatial memory have also been observed, such as an impairment in reversal learning following classic conditioning for rats fed a high fat diet supplemented with high concentrations of either glucose or sucrose for 3 months (Kanoski et al., 2007), in non-spatial reference and working memory after 30 days of exposure (Kanoski and Davidson, 2010), in fear conditioning (Xu and Südhof, 2013; Spencer et al., 2017), and in novel object recognition (Gainey et al., 2016) or location (Heyward et al., 2012). Together, these findings confirm the hypothesis that high-energy diets alter aspects of hippocampal and cortical function—as well as other brain regions such as the amygdala—to affect learning and memory; a handful of studies, however, failed to show any changes in some types of learning and memory tasks with these diets (Mielke et al., 2006; McNeilly et al., 2011; Heyward et al., 2012). A recently published meta-analysis indicates that in humans diets with high levels of saturated fats, and particularly, those with both saturated fat and added sugars, negatively impacted measures of hippocampal function, such as learning and memory or hippocampal volume (Taylor et al., 2021).

Alterations in the function of the hippocampus are also suggested by studies that observed changes in the expression levels of genes important for learning and memory, although the direction of these changes varied and may thus depend on the specific type and length of dietary exposure. For example, the levels of the Brain Derived Neurotrophic Factor (BDNF) were found to be lower (Kanoski et al., 2007; Park et al., 2010; Pistell et al., 2010), higher (Sharma and Fulton, 2013), or unchanged (Pistell et al., 2009; Heyward et al., 2012; Beilharz et al., 2014) with exposure to high fat chow or high fat and high sugar human foods. In addition to variation in BDNF levels with these diets, other researchers also measured higher expression of pro-inflammatory genes, such as the Tumor Necrosis Factor α (TNFα; Pistell et al., 2010; Beilharz et al., 2014; Boitard et al., 2014), InterLeukin-6 (IL-6; Pistell et al., 2010; Boitard et al., 2014), InterLeukin-1-β (IL-β; Pistell et al., 2010; Boitard et al., 2014; Sobesky et al., 2014), chemokine monocyte chemoattractant protein 1 (MCP-1), and other markers of microglia activation (Pistell et al., 2010). Other metabolic markers of neuroinflammation, such as an increase in acyl-CoA and reactive oxygen species, malondialdehyde, and Nitric Oxide, were also detected in the hippocampus, cortex, brainstem, and cerebellum of rodents fed high fat diets (Ha and Redmond, 2008; Posey et al., 2009; Wang et al., 2020). Neuroinflammation and lower numbers of dopaminergic neurons in the Substantia Nigra- phenotypes reminiscent of those that occur in Parkinson’s disease—were uncovered in mice exposed to a high fat diet for 5 months after weaning (Kao et al., 2020). In addition to inflammation, hallmarks of insulin resistance—such as higher expression of the insulin receptor and lower levels of glucose transporters—were also measured in the hippocampus, as well as a reduction in synaptic scaffolding proteins and in the levels and phosphorylation of activity-regulated genes such as the Activity-regulated cytoskeleton-associated protein 1, c-Fos and Extracellular-signal Regulated Kinase (ERK; Ha and Redmond, 2008; Posey et al., 2009; Arnold et al., 2014; Kothari et al., 2017); this interplay between metabolic changes, memory, and disease has been recently reviewed in Garcia-Serrano and Duarte (2020). Early life exposure to sugar and memory alterations have also been linked to microbiome changes (Noble et al., 2021).

### Affective Behaviors

Calorie-dense diets and BMI have also been associated with anxiety and depression in humans (Adan et al., 2019). Studies in rodents suggest the performance on behavioral tests that are used as a proxy for these conditions—like the open field assay, the elevated plus maze, and the forced swim assay—is influenced by exposure to a high fat and sugar diet, alone or in combination. For example, mice on a high fat diet for 12 weeks or rats on a high fat and high fructose diet for 8 weeks spent less time in the open arms of the elevated plus maze, more time immobile in the forced swim test, and had shorter social interactions (Sharma and Fulton, 2013; Gancheva et al., 2017); interestingly, an increase in anxiety phenotypes was also present in rats only after a shorter, 3 weeks exposure to a high fat and high glucose/sucrose diet (Peris-Sampedro et al., 2019). However, consumption of a high fat and sugar diet after weaning for 10 weeks alleviated anxiety-like behaviors caused by exposure to early life stress in rats, with no accompanying changes in spatial memory or object recognition (Maniam et al., 2016), showing that diet can interact with other types of environmental stressors. That said, in rats consumption of a high fat diet for 6 weeks did not worsen the effects of chronic mild stress, although the behavioral phenotypes of stress and exposure to a high fat diet were similar (Aslani et al., 2015). An increase in anxiety phenotypes with diet correlated with a higher expression of BDNF and the immediate early gene c-Fos in the striatum (Sharma and Fulton, 2013; Nguyen et al., 2017), lower dopamine levels (Nguyen et al., 2017), and lower expression of the RAC-α serine/threonine-protein kinase Akt, while their amelioration after early life stress reflected higher glucocorticoid protein levels (Maniam et al., 2016).

### Summary

Together, these studies suggest that in rodents diets high in fat and sugar, alone or a combination (either as chow or as human food), alter neural signals that regulate energy balance, satiation and satiety, and reward responses to food; these alterations promote food intake and, with prolonged consumption, weight gain and metabolic disease. A high fat, high sugar diet, alone or in combination, also leads to changes in taste and smell in rats and mice, which may contribute to the deregulated food intake observed. The observed alterations in gene expression and neuropeptide and neurotransmitter signaling also play an important role in these behavioral phenotypes. In rodents consumption of these diets—although composition and length of exposure vary greatly—is generally associated with impairments in different types of memory and with anxiety-like behaviors (Figure 1). While many of these effects have been observed in humans with high BMI, it remains unclear whether diet composition, caloric density, or a combination of both, contributes to them; further, whether they are caused by diet, or are instead a consequence of metabolic dysfunctions that develop with excess body weight and obesity remains largely unknown. The underlying mechanisms also remain elusive, but a large number of studies have pointed towards neuroinflammation, with higher levels of proinflammatory cytokine, microglia activation, and changes in the expression of immediate early genes and synaptic proteins, all of which are involved in the establishment and consolidation of memories; new studies are also investigating the role of the microbiome (Figure 1, purple).

## The Effects of High Fat Diet on Zebrafish Behaviors

*"Fish are friends, not food."—Bruce the Shark from Finding Nemo©*

In the last decade, the zebrafish *D. rerio* has emerged as a new model organism to study metabolic diseases (Williams and Watts, 2019). Thanks to its easy maintenance, high fecundity, and powerful genetics, this organism has been used to study the effects of genes associated with human BMI and obesity, and to discover new genes involved in fat regulation and body weight (Seth et al., 2013; Choi et al., 2021). More recently, researchers have developed several diet-induced obesity paradigms and studied the effects of these dietary environments on behavior (Figures 1, 2). To increase the caloric content of food, zebrafish are fed Artemia, a live prey with high fat content, or standard fish chow supplemented with lard or egg yolk (Williams and Watts, 2019). Feeding zebrafish a high fat diet based on Artemia ad libitum for 4 or 8 weeks increased BMI (measured as g weight/cm^2^ length), plasma TAGs, and hepatic fat accumulation in both males and females (Oka et al., 2010; Ghaddar et al., 2020). At 8 weeks, there was a dysregulation in the expression of ~150 genes in the adipose tissue, including those involved in inflammation and lipid metabolism (Apolipoprotein H, *il6* and IL-1β, Sterol regulatory element binding transcription factor 1, peroxisome proliferator-activated receptor α/γ, nuclear receptor subfamily 1 group H member 3 and Leptin; Oka et al., 2010). Fish fed this high fat diet for 4 weeks presented substantial leakage of the blood brain barrier, as revealed by injection of Evans blue dye, as well as increased expression of inflammatory markers, such as IL-1β, *il6*, pro-inflammatory transcription factor *nfkb*, and tumor necrosis factor α (TNF-α; Ghaddar et al., 2020). Consistent with this, the authors observed a higher number of activated (amoeboid) microglia cells in the hypothalamus and the ventral telencephalon. Neuroinflammation is a known disruptor of adult neurogenesis, and in fact the authors found that in several parts of the brain, including the ventral and dorsal telencephalon, the anterior part of the preoptic area, the periventricular pretectal nucleus, and two caudal hypothalamic regions, showed lower cell proliferation as measured by Proliferating Cell Nuclear Antigen staining. Diet-induced obesity also correlated with increased inactivity, but not with distance traveled, although the connection between these behavioral changes and the molecular and cellular deficits measured was not established (Ghaddar et al., 2020). A second study that used a different high fat diet version by supplementing the standard fish chow with two different concentrations of chicken egg yolk (16.9% or 21.1% compared to the standard diet concentration of 6.5%) for 2 weeks, reported significantly lower exploratory behavior without any other changes in locomotion in the novel tank test in the high fat diet fish, suggesting an increase in anxiety-like behaviors (Picolo et al., 2021). Interestingly, these high fat diet fish also exhibited a higher duration, but not number, of aggressive episodes, without any changes in social preference; they also presented deficits in aversive memory formation, and those fed the highest concentration also showed poorer retention of the memory. However, the causes of such behavioral abnormalities were not elucidated and it was not clear whether they arose from the increase in body weight associated with the high fat diet, or the higher fat content itself (Picolo et al., 2021). Cognitive function, as measured by the active avoidance test, was also impaired in zebrafish fed a high fat diet composed of 20% chow and 80% lard for 11 weeks. RNAseq revealed about 100 genes with changes in mRNA abundance in the telencephalon, including those that are involved in cognitive function in mammals, such as Postsynaptic Densities 95 (PSD95), BDNF, Nuclear factor erythroid/p45-related factor, and Presenilin 1 and Presenilin 2 (Meguro et al., 2019); interestingly, this diet leads to a small ~10% increase in energy intake but no changes in body weight and fat levels, suggesting that perhaps the effects on cognition were due to diet composition and not higher caloric intake.

### Summary

Together these studies in fish show that different high fat diet regimens can alter cognitive function, learning and memory, aggression, and anxiety behaviors (Figure 1); however, whether these alterations are caused by inflammation or reduced neurogenesis is unclear, as well as whether they arise as consequences of higher BMI or diet composition.

## The Effect of High Fat and High Sugar Diets on *D. Melanogaster* Behaviors

“You buttered your bread. Now sleep in it!”*—Jiminy Cricket from Pinocchio©*

Over the last century, our understanding of biology and medicine has been transformed by the use of *Drosophila melanogaster* as a model organism. The short generation cycle, exquisite genetic tools, and the remarkable conservation of disease genes and pathways, have made the fly an ideal model for studies in a myriad of fields, including cell biology, genetics, and aging. More recently, the fly has also been used to understand the molecular mechanisms through which diet affects body and brain physiology (Musselman and Kühnlein, 2018). Dietary manipulations in flies are done by adding different percentages of different sugars (most commonly 20–30% sucrose, fructose, glucose) or fats (10–20% lard, coconut oil, or other fats) to their standard food and can be done both during development (larvae) and in adults; here we will discuss only adult exposure studies.

Adult flies fed a high fat or high sugar diet for 7–14 days have higher lipid levels and altered insulin and glucose homeostasis (Birse et al., 2010; Musselman et al., 2011; May et al., 2019; Wilinski et al., 2019; Lourido et al., 2021), as well as changes in the levels of hundreds of metabolites, including amino acids, 1-Carbon metabolism, and nucleotides, organic acids, and overall carbon-nitrogen balance, depending on the type and length of diet exposure (Heinrichsen et al., 2014; Wilinski et al., 2019; Figures 1, 2). These metabolic phenotypes reflect hallmarks of metabolic syndrome, and despite differences in anatomy compared to vertebrates, they contribute to defects in heart contractility and structure (Birse et al., 2010; Na et al., 2013) and in the function of the malpighian tubules; they also shorten lifespan (Na et al., 2013; Jung et al., 2018) and lower the resistance to other metabolic stresses (Heinrichsen and Haddad, 2012). The similarities in metabolic and physiological outcomes are perhaps not surprising considering that they are the result of the activation of conserved nutrient-sensing pathways, such as insulin-Target of Rapamycin (TOR; Birse et al., 2010; Li et al., 2010), hexosamine biosynthesis (Na et al., 2013; May et al., 2019), and Janus Kinases-Signal Transducer and Activator of Transcription proteins (JAK-STAT; Yu et al., 2018; Lourido et al., 2021). As in mammals, flies fed with these diets also show alterations in genes involved in immune response, inflammation, metabolism, neural signaling, synaptic function, and sensory perception in the brain and specific populations of neurons (Hemphill et al., 2018; Jung et al., 2018; May et al., 2019; Stobdan et al., 2019; Vaziri et al., 2020); some of these variations in gene expression have been causally linked to neural and behavioral changes, while others only remain correlated. Differences in the levels of neurotransmitters and neuromodulators have also been observed with both high sugar (Wilinski et al., 2019) or high fat diets, including a decrease in serotonin and octopamine (Meichtry et al., 2020), and an increase in inflammatory factors homolog to transforming growth factor-β (Hong et al., 2016). As with rodents, many different behaviors have been phenotyped in response to dietary manipulations in flies, including feeding and reward, taste and olfaction, sleep, and mood, while less is known about their effects on learning and memory, although many of the genes impacted by sugar are known to play a role in cognition.

### Ingestive Behavior

In adult flies, consumption of a high fat and high sugar diet (2% palmitic acid and 10% sucrose) for 7 and 14 days was associated with lower olfactory sensitivity to both appetitive and aversive odors, and differences in olfactory choice preference, presumably due to changes in the mRNA levels of olfactory receptor and olfactory binding proteins in the antenna (Jung et al., 2018). Changes in behavioral olfactory responses to some, but not most odors, as well as the head mRNA levels of genes involved in sensory perception, metabolism, and motor function, were also revealed in flies fed a 20% coconut oil diet for 7 and 14 days (Rivera et al., 2019). In addition to olfactory deficits, studies have shown that in flies exposure to different amounts of sugar in the diet (10, 20, and 30% sucrose) also dulled sweet taste sensation by lowering the responses of the sweet taste cells to sweet stimuli (May et al., 2019; Vaziri et al., 2020; May and Dus, 2021). This chemosensory plasticity developed early (2–3 days) after dietary exposure and also occurred in flies fed high glucose or high fructose diets, but not in animals fed a sweet but not calorically-dense diet (sucralose) or a lard diet equi-caloric with the high sugar diets; further, genetically obese animals showed no changes in taste sensation, while obesity-resistant flies experienced taste plasticity. These findings suggest that sugar metabolism played a key role in altering the neural responses to sweetness independently of weight gain or fat accumulation. Indeed, the authors found that the hexosamine biosynthesis pathway enzyme O-GlcNAcTransferase acted in a cell autonomous way to dull the responses of the taste cells to sweet (May et al., 2019). A followup study showed that sugar metabolites induce taste plasticity by remodeling the transcriptome of the sweet sensing neurons (Vaziri et al., 2020); specifically, the authors uncovered that the epigenetic silencer Polycomb Repressive Complex 2 (PRC2) suppressed a neurodevelopmental program required for neural plasticity by changing its binding to chromatin in a diet-dependent way (Vaziri et al., 2020). Of note, another group uncovered that dietary sweet taste plasticity also engaged a signaling pathway that activated the transcription factors cAMP-response element binding protein (CREB) and Peroxisome proliferator-activated receptor-γ coactivator 1-α (PGC1α; Wang et al., 2020).

These changes in taste function have been causally linked to alterations in food intake and meal size. Similarly to humans and rodents, consumption of energy-dense diets promotes higher intake in *D. melanogaster*. In flies fed a high sucrose diet the size and duration of meals was doubled that of animals on a control diet; these changes also occurred in obese resistant flies, showing that they are caused by diet and not weight gain. However, correcting the activity of the sweet taste neurons resulted in normal feeding and meal size and protected flies from diet-induced obesity (May et al., 2019). To understand how diet-induced taste plasticity affected intake and meal size, the authors examined whether the responses of dopaminergic neurons to sugar were altered by a high sugar diet. In mammals and insects, the neuromodulator dopamine is secreted during eating depending on the taste and nutrient properties of foods (Huetteroth et al., 2015; Yamagata et al., 2015; Tellez et al., 2016; Thanarajah et al., 2019); these signals are used in downstream circuits to impart saliency and modulate learning and memory. Exposure to a high sugar diet, but not to an equi-caloric high fat diet, for 7 days dulled and delayed the responses of a subset of dopaminergic neurons to sugar; these deficits, however, were corrected in animals where sweet taste function was restored pharmacologically. Further, in high sugar diet fed flies, activating the dopaminergic neurons with optogenetics restored normal meal size, feeding rate, and overall feeding, as well as protected them from weight gain (May et al., 2020). This suggests that changes in dopamine signaling due to diet originate in part from alterations in sensory perception and that they drive larger meals and intake.

### Activity, Sleep, and Courtship Behavior

Dopaminergic neurons are also involved in movement and sleep, and indeed, flies fed two different types of high fat diets had impaired climbing activity and phototaxis memory (Jung et al., 2018; Rivera et al., 2019). Flies on a high fat diet also had a greater walking speed upon fasting, along with a shorter latency to food seeking, but no difference in energy expenditure (Huang et al., 2020). This was attributed to the higher activity of the octopaminergic neurons on a high sugar diet due to elevated protein levels of the Adipokinetic hormone (AKH) Receptor; this enhanced the effects of AKH, a hunger hormone functionally analog to glucagon that impacts the animal’s overall activity. A high sucrose diet was also associated with changes in sleep architecture, specifically fewer sleep bouts of longer duration; this occurred after one-two of exposure to the diet, and even in genetically lean flies, suggesting that it was due to diet composition, not fat accumulation (Linford et al., 2012). Besides eating, sleeping, and climbing, other motor and exploratory behaviors are also affected by high fat diets in flies. In a forced swim test flies on the hydrogenated vegetable fat or a lard diet had higher total immobility time, lower total swimming compared to flies on a control or high sugar diet (Meichtry et al., 2020). These animals spent more time on the dark side of the chamber and groomed more; they also showed higher aggressive behaviors, such as chasing and wing raising, when fed the hydrogenated vegetable diet compared to the lard or control diets. Interestingly, the authors observed that the levels of octopamine and serotonin were lower in the high fat diet conditions, which is interesting considering that in humans these neurotransmitters have been linked to anxiety and depression, conditions known to be influenced by BMI. Finally, a high fat diet also changed mate preference and mating behavior in *D. melanogaster*. Specifically, male flies mated less with females fed a high fat diet and more with those on a high sugar diet, regardless of which diet the males were on. Of note, female flies on the high fat or high sugar diets had higher mating receptivity and mated faster on average, compared to control diet females (Schultzhaus et al., 2017). However, males on a 30% (but not 15%) high fat diet courted females less and took longer to mate, suggesting that this diet negatively impacted male attractiveness to control diet-fed females (Schultzhaus et al., 2018). Some of these differences in courtship could be attributed to alterations in cuticular hydrocarbons—insect pheromones that play a crucial role in this process—but not to variations in the male courtship song (Schultzhaus et al., 2018).

### Summary

Together, studies in *D. melanogaster* flies argue that diets supplemented with high amounts of sugars and fats promote food intake and influence behaviors like sleep, aggression, anxiety, courtship, and depression (Figure 1). It is particularly interesting to reflect on the role that diet-driven sensory changes play in these behavioral changes, and indeed, several of these studies drew a causal link between sensory function, specifically taste, and alterations in meal size and total feeding and sleep. In contrast, relatively little is known about how these diets influence neuroendocrine signals that control energy homeostasis or cognition, although genes involved in these processes were changed by diet. Importantly, some of these studies have used the power of *D. melanogaster* genetics to uncouple the effects of dietary exposure from weight gain and revealed that sensory plasticity, larger meal size, and sleep are directly affected by diet composition and not excess fat mass or higher caloric intake. Finally, transcriptomics studies suggest that a high fat diet also influences the expression of genes involved in immunity, infection, nutrient storage, and signaling in heads (Hemphill et al., 2018) in manners similar to those described in rodents. Interestingly, some changes in gene expression, including those involved in stress response, lipid and carbohydrate metabolism, glycosidase activity, and fatty acid metabolism, are sexually dimorphic (Stobdan et al., 2019).

## Concluding Remarks and Open Questions

*“You and I are a team. There is nothing more important than our friendship.”*

*Mike Wazowski, Monsters Inc*.

In humans, diets high in saturated fat and added sugars promote higher calorie intake, weight gain, and the development of the metabolic syndrome and chronic diseases. These effects also occur both in large mammalian models like dogs and pigs and in both traditional and non-traditional small laboratory model organisms, suggesting broad conservation in the molecular and cellular pathways that underlie these conditions. The consequences of these diets are not only limited to the body, but also extend to the brain, and indeed in humans, alterations in the ingestive and motivated behaviors, sensation, cognition, mood, and sleep have been observed with energy-dense diets or high BMI. As we have highlighted here, similar phenotypes have also emerged in vertebrate and invertebrate animal studies, indicating that nutrient status may engage common pathways even in the nervous system. The most similar effect of these diets across models is on feeding behavior. There is indeed no doubt that ingestive behavior is profoundly altered by the dietary environment, as evidenced by the rodent, fish, and fly studies discussed above. Consumption of a high fat, high sugar, or a combination of both diets, leads to higher intake in all models, including bigger meals in rodents and flies. Alterations in chemosensensation, specifically taste and smell, may desensitize animals to sensory-enhanced and sensory-induced satiety, leading to larger meals and higher intake. The chemosenses also play an important role in nutrient prediction and expectations by signaling through the dopaminergic system. Changes in dopamine levels, transduction, and transmission with diet and high BMI could arise from both alterations in chemosensation and also be the result of the cell-autonomous effect of nutrients on these circuits. Deregulated ingestive behaviors could further originate from changes in the responses to satiety hormones and peptides, and indeed, their levels and efficacy are decreased with diet-induced obesity in rodents. Deficits in learning and memory also occur with exposure to high-energy diets in rodents and fish, as well as changes in behaviors linked to anxiety and depression in rodents and flies. Thus, calorie-dense diets have profound effects on behavior across model organisms (Figure 1).

Despite this consensus, however, there are a number of challenges in these studies that limit our ability to understand the extent of the relationship between diet, brain, and behavior. First, very few experiments were carried out to show that dietary manipulations or obesity caused changes in behavior. Second, only a few studies have monitored and measured the effects of diet on neural activity in the attempt to link behavioral alterations with neural phenotypes. Moreover, when molecular studies were performed, these were done in whole brain regions, rather than using techniques to measure changes in specific circuits. Importantly, most of these studies used male animals, and only a few examined the effects of diet on females. Addressing these limitations should be possible by using transgenic animals and recently-developed molecular, imaging, and opto/neurogenetic techniques, to assess, monitor, and control the effects of diet on neural physiology and behavior. Finally, heterogeneity in diet regiments and exposures, as well as the models’ unique physiology and dietary niche, may lead to inappropriate comparisons to humans and to incorrect conclusions. Despite these limitations and caveats, however, these studies do suggest that components of the modern dietary environment have an influence on behavior; what remains largely unknown, however, is how this occurs.

### Open Questions

**Figure 3:**
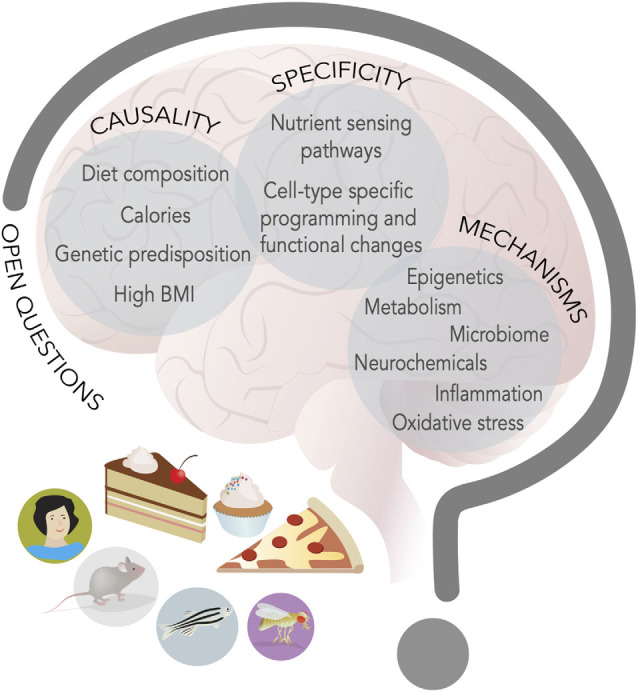
New food for thought. Research in model organisms and humans has advanced our knowledge of the interplay between energy-dense diets and behavior, but at least three main questions remain open (capital letters); hints to possible mechanisms (listed inside circles) have emerged and studies on these topics will define the next decade of research in the neuroscience of nutrition field.

The first open question is the etiology of these behavioral and neural changes: are they the result of diet composition, high caloric density, excess body weight, and fat mass, or peripheral or central metabolic changes (Figure 3)? The most likely explanation is that all these aspects play a role, but that the timing and the consequences may indeed be quite different. For example, research in flies and rodents suggests that diet composition, rather than extra calorie or metabolic syndrome, contributes to taste plasticity, but phenotypes worsen with dietary exposure, which suggests that physiological changes due to metabolic syndrome may also have an influence. These broad metabolic changes could play a particularly important role in the neuroinflammation that seems to characterize cognitive and mood dysfunctions. Model organisms like flies and zebrafish are ideally suited to tackle this question, because these factors can be manipulated genetically, for example by exposing animals that are obesity-resistant or genetically obese to different diets.

A second outstanding question is how the same diet leads to such broad effects on the nervous system. Are there specific nutrient sensing pathways that are engaged in different neural and neuronal cell types or do a handful of pathways give rise to diverse effects (Figure 3)? It certainly seems that a few of the same nutrient-responsive cellular pathways and enzymes—like mTOR, hexosamine biosynthesis, pentose phosphate, and AMPK, GCN2, Akt/PI3K, SREBP, PPAR, and glucokinase to name a few (Efeyan et al., 2015)—mediate many of the effects of diet on cellular physiology. The presence of receptors and transporters tuned to nutrients and metabolites or hormones provides specificity for which cells are engaged by diets, but the outcome of their activation is different depending on the cell types. What provides this outcome specificity? We know very little about this question, but one possibility is that the outcome is linked to the neural cell-type specific gene batteries (Deneris and Hobert, 2014). Each cell type expresses a unique combination of transcription factors that shapes the expression of genes and the biophysical and cellular properties of that cell, which limits the outcome available to nutrient-sensing pathways (Deneris and Hobert, 2014). As such, in a dopaminergic neuron, high sugar or fat may repress expression of TH, while in a hippocampal neuron it may silence BDNF, and in another cell type alter the translation or halflife of a transcript *via* RNA methylation. Further, even when the metabolic consequences may be shared across different cell types, as in the case, for example, of mitochondrial dysfunction, they may still give rise to separate results because of differences in cell energy requirements or function. For example, mitochondria are involved in ATP production, redox balance, and Radical Oxygen Species (ROS) disposal, and neurons may have different sensitivities to their dysfunction and thus be more or less affected by it. Measuring the effects of diet on specific cell types or circuits, integrating this information with screens in model organisms, and carrying out followup studies in preclinical models, will all be critical to answering this question. Importantly, it will be essential to also consider the question of nutrient balance, both by investigating the effects of low-fat and sugar diets, but also by examining the role that high levels of these macronutrients may play on the balance of others; for example, in humans, high fat and high sugar diets are often protein poor, and the so the effects on behavior could both be the result of high levels of sugar and fat and of low amino acids.

A third, open question is that of mechanisms. What are the molecular, cellular, and neural mechanisms through which diets change neural function and behavior (Figure 3)? Nutrients and metabolites themselves can influence cell physiology by directly or indirectly altering gene expression. Indeed, most metabolites are used as cofactors for DNA, RNA, and protein modifying enzymes, or can modulate their activity or association to DNA, chromatin, and RNA. Variations in metabolite levels because of diet can sculpt cells’ responses to the environment and these effects can be long-lasting and persist even after the dietary environment has dissipated; they can also reshape or weaken the identity of cells (Vaziri and Dus, 2021). Changes in nutrient levels can also influence the abundance and secretion of neurochemicals, as most neurotransmitters are byproducts of central or amino acid metabolism (Dai et al., 2020). Further, many transporters and receptors are sensitive to nutrient amounts or can be modified by intracellular concentrations of metabolites (i.e., K_ATP_ channels, Transient Receptor Potential channels) and thus modify the responses of cells to stimuli (Raka et al., 2019). More severe metabolic changes due to long-term, chronic consumption of some diets, excess fat mass, or neuroendocrine alterations, can also stress cellular organelles and compartments, such as mitochondria, the ER, lysosomes, or Golgi, impair cell function, and promote pro-inflammatory states that, in turn, negatively affect cell physiology (Castelli et al., 2019; Sekine et al., 2021). Importantly, fluctuations in metabolites, nutrients, and neurochemicals may shift or be the result of shifts in the composition of the microbiome, which has been recognized as an important force in shaping neural function and behavior (Zangara and McDonald, 2019).

The answers to these challenging questions will require reaching and integrating across disciplines and, importantly, across model organisms. As Krogh’s principle suggests, each animal model has unique strengths and advantages that lend themselves to dissecting different aspects of these questions, as well as using other, both established and yet-to-be-established, models. While it may be hard for any single laboratory to test hypotheses among different models or carry out complex techniques in different fields, collaborations across labs could ensure that we collectively find the answers to these questions. Thus, the next decades of research in the neuroscience of nutrition will require teamwork and lots of it. While this teamwork will solve the mystery of diet and the brain, and, perhaps, lead to new pharmaceutical and public health interventions that treat or curb diseases, it will not, alone, solve many of the issues that originate these changes. Indeed, unequal access to fresh food across cities and countries, as well as the lack of transparency in food labels, sourcing, and marketing, profoundly contribute to the burden of malnutrition and health disparities, both domestically and globally (Perez-Escamilla et al., 2018). Solving this question will require teamwork among scientists and scholars in other disciplines, such as sociology, urban planning, economics, business, and law, in addition to the efforts of legislators, governments, nonprofits, grassroots organizations, and policymakers worldwide. These synergistic partnerships will help empower communities with a food environment that supports healthy aging, encourages mindful eating, and promotes mental and physical wellbeing.

## Author Contributions

MD and MS wrote and edited the manuscript together. All authors contributed to the article and approved the submitted version.

## Conflict of Interest

The authors declare that the research was conducted in the absence of any commercial or financial relationships that could be construed as a potential conflict of interest.

## Publisher’s Note

All claims expressed in this article are solely those of the authors and do not necessarily represent those of their affiliated organizations, or those of the publisher, the editors and the reviewers. Any product that may be evaluated in this article, or claim that may be made by its manufacturer, is not guaranteed or endorsed by the publisher.
